# Targeted Molecular Imaging Probes Based on Magnetic Resonance Imaging for Hepatocellular Carcinoma Diagnosis and Treatment

**DOI:** 10.3390/bios12050342

**Published:** 2022-05-17

**Authors:** Dongxu Zhao, Jian Cao, Lei Zhang, Shaohua Zhang, Song Wu

**Affiliations:** 1 Department of Urology, The Third Affiliated Hospital of Shenzhen University (Luohu Hospital Group), Shenzhen 518000, China; 20204232031@stu.suda.edu.cn; 2 Department of Interventional Radiology, The First Affiliated Hospital of Soochow University, Suzhou 215006, China; 3 Department of Gastroenterology, The Affiliated Suzhou Hospital of Nanjing Medical University, Suzhou Municipal Hospital, Suzhou 215006, China; caojiansz@njmu.edu.cn; 4 Center of Interventional Radiology & Vascular Surgery, Department of Radiology, Zhongda Hospital, Medical School, Southeast University, Nanjing 210009, China; 5 Guangzhou Institutes of Biomedicine and Health, Chinese Academy of Sciences, Guangzhou 510530, China; 6 Department of Urology, The Affiliated South China Hospital of Shenzhen University, Shenzhen University, Shenzhen 518000, China

**Keywords:** molecular imaging probes, magnetic resonance imaging, nanomaterials, hepatocellular carcinoma

## Abstract

Hepatocellular carcinoma (HCC) is the sixth most commonly malignant tumor and the third leading cause of cancer-related death in the world, and the early diagnosis and treatment of patients with HCC is core in improving its prognosis. The early diagnosis of HCC depends largely on magnetic resonance imaging (MRI). MRI has good soft-tissue resolution, which is the international standard method for the diagnosis of HCC. However, MRI is still insufficient in the diagnosis of some early small HCCs and malignant nodules, resulting in false negative results. With the deepening of research on HCC, researchers have found many specific molecular biomarkers on the surface of HCC cells, which may assist in diagnosis and treatment. On the other hand, molecular imaging has progressed rapidly in recent years, especially in the field of cancer theranostics. Hence, the preparation of molecular imaging probes that can specifically target the biomarkers of HCC, combined with MRI testing in vivo, may achieve the theranostic purpose of HCC in the early stage. Therefore, in this review, taking MR imaging as the basic point, we summarized the recent progress regarding the molecular imaging targeting various types of biomarkers on the surface of HCC cells to improve the theranostic rate of HCC. Lastly, we discussed the existing obstacles and future prospects of developing molecular imaging probes as HCC theranostic nanoplatforms.

## 1. Introduction

Liver cancer is regarded as a global health issue, with its prevalence increasing in recent years, and it has risen to become the fourth most common cause of cancer-related death globally [1,2]. Hepatocellular carcinoma (HCC), the most prevalent kind of primary liver cancer, usually arises in the context of chronic liver disease, with hepatitis B virus (HBV) or hepatitis C virus (HCV) infection, alcohol abuse, and nonalcoholic fatty liver disease [3,4]. At present, the five-year survival rate of HCC is still unsatisfactory [5,6]. The five-year survival rate of HCC patients from China has been reported to be only 12.1%, presenting a bleaker future than in other countries with 30.1%, 27.2% and 17.4% for Japan, Korea and America, respectively [7]. The main reason for the poor long-term prognosis is that most patients are effectively unable to access early diagnosis and treatment. The concealment of HCC is a particularly problematic issue [8], owing to the fact that individuals in the early stage are asymptomatic with well-preserved liver function, meaning that it is difficult for them to detect that something is wrong. On the other hand, patients in the early stage usually have a light tumor burden and small tumor diameter, which is difficult to identify and causes difficulties in clinical diagnosis [9]. By the time most patients are diagnosed, the optimal treatment period will have passed. Therefore, improving the early diagnosis rate of HCC is urgently required to reduce the mortality of patients with HCC [10,11].

Currently, the clinical diagnosis of HCC still depends on imaging methods. Among the various imaging methods, magnetic resonance imaging (MRI) is the standard scheme for the diagnosis of HCC according to international guidelines [12,13,14]. Compared to other imaging methods, MRI possesses some advantages such as good safety with the absence of ionizing radiation, high spatial resolution, great soft tissue contrast, and a better depth of penetration, making MRI an excellent diagnostic tool in the identification of HCC [15,16,17]. However, due to the limited specificity of MRI in diagnosing early tiny HCC nodules, its accuracy in detecting early stage HCC is not ideal [18,19,20,21], especially for some minor lesions (HCC lesions < 2 cm) [22], making it difficult to distinguish them from other benign nodules and leading to false-negative results [19,23]. According to the result from a retrospective study, the diagnostic performance of gadoxetic acid enhanced MR imaging for the identification of HCC, indicating that for a size of less than 1 cm it is poor, with a mean sensitivity of 46% for tiny malignant hepatic nodules diagnosis [21]. Even so, there is no doubt that MRI still has unique advantages and great potential in the diagnosis of HCC, thus, how to improve the sensitivity and specificity of MRI in the diagnosis of early small HCC and malignant nodules is a critical issue that requires study.

Molecular imaging, first systematically introduced in 1999 by Weissledertl, is a technique for noninvasive visualization and quantitative description at the genetic, molecular, cellular, or even at the tissue and organ levels in vivo [24]. In recent years, advances in biomaterial technology and biomarker assay techniques have induced the development of novel biomarkers and the invention of numerous molecular-imaging agents [25,26]. These achievements have been applied for the development of novel probes for the targeted imaging of various diseases [27,28]. Based on this, some studies suggest combining molecular imaging with MRI to improve the accuracy of the early diagnosis of HCC. These experiments are mainly concerned with the cellular and animal levels, but provide significant promise and encouraging outcomes. Prior to developing more advanced techniques for the accurate and efficient diagnosis of HCC, a comprehensive understanding of the recent advances is required. Hence, this review provides a timely summary of the previous experimental studies of MRI-based targeted molecular-imaging probes in the theranostics of HCC. Although many studies are still in the experimental stage, the promising results and ideas they present may provide valuable strategies for future clinical applications. In this review, we firstly introduce the reasons for selecting the targeted-imaging strategy and its advantages compared with the traditional methods, and discuss the common targets for HCC. Secondly, we summarize the types of commonly used ligands and their merits and defects. Thirdly, theranostic applications of HCC-receptor-targeted molecular-imaging probes based on MR imaging are reviewed. Finally, we conclude by outlining the existing obstacles and future prospects for developing molecular-imaging probes for HCC theranostic nanoplatforms.

## 2. Advantages of Targeted Delivery Strategy and Common Targets

To accurately transport molecular probes containing paramagnetic substances into tumor tissues, and to achieve early diagnosis and even combined treatment in the body, the main challenges are as follows: (1) making probes that are concentrated in the tumor site, and (2) retaining and avoiding the impact of blood flow. The traditional delivery strategy faces a series of abnormal vascular-system problems in solid tumors such as a large number of new lymphatic vessels and blood vessels, ineffective lymphatic drainage, high permeability and reflux disorder [29,30]. The abnormal structure can lead to the long-term accumulation of drugs in tumors [31,32], a phenomenon that was named the enhanced permeability and retention effect (EPR effect) by Gerlowski et al. in 1986 [33,34]. Based on these properties, passive drug delivery can be achieved [35]. This passive delivery strategy has become a major player in recent decades for cancer treatments. Its primary advantage is its simplifying of the operation. As passive nanodrug administration is mainly based on differences between the physiological qualities of the liver and the pathological aspects of HCC, the biocompatibility surface modification of nanodrugs needs to be considered to ensure that they can participate in the blood circulation for as long as possible and eventually reach the tumor site, rather than being swallowed by the reticuloendothelial system [36]. However, the disadvantages of passive drug delivery cannot be overlooked. Firstly, the whole process depends largely on the EPR effect of the tumor itself. If the EPR effect of the tumor is not clear, this method becomes ineffective. Secondly, the permeability of each part of the whole tumor is not uniform, making drug distribution over the whole tumor-tissue area difficult, which may lead to long-term recurrence due to tumor residue. Thirdly, nanodrugs may remain deposited in certain natural liver tissues, thereby reducing therapy efficacy [35] and making the entire treatment process difficult to control. Finally, the absence of targeting particularly effective HCC cells leads to the limited internalization of cancer cells [36].

**Figure 1:**
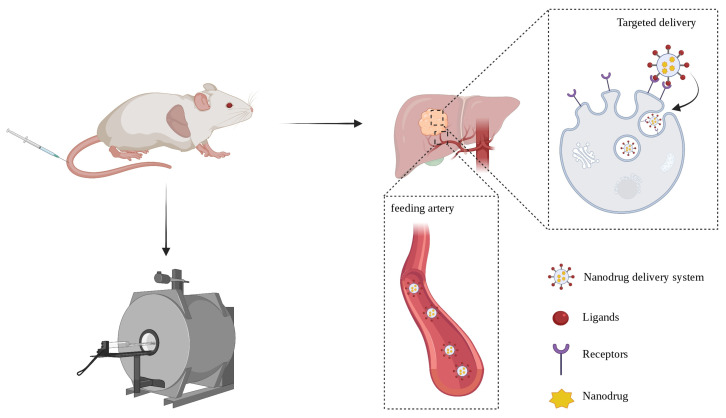
The schematic diagram of nanodrug targeted delivery.

Another delivery strategy is active targeted delivery, which is discussed in this review. By exploring the biological characteristics of cancer cells, various types of ligands such as antibodies, proteins, peptides, nucleic acids and receptors can be affixed to the surface of nanocarriers to target cancer cells and cellular internalization [37]. Active-targeting modification is more complex than passive delivery, and its advantages are also clear. This administration method avoids the nonspecific uptake of normal cells and the related toxicity to healthy cells. The carrier can also be modified to achieve the purpose of responsive release, so that nanodrugs can be released under specific conditions to kill cancer cells with precision. The targeted drug dose is smaller and individual tolerance improves [38]. A comparison of the characteristics of passive delivery and active targeted delivery is provided in Table 1. In practical operation, the ligand can be connected to the surface of the nanodrug through corresponding modifications, which is helpful for intracellularization, and the paramagnetic substances carried in it can be finely detected by MRI for the diagnosis of HCC [39], for which the process is depicted in Figure 1.

**Figure 2:**
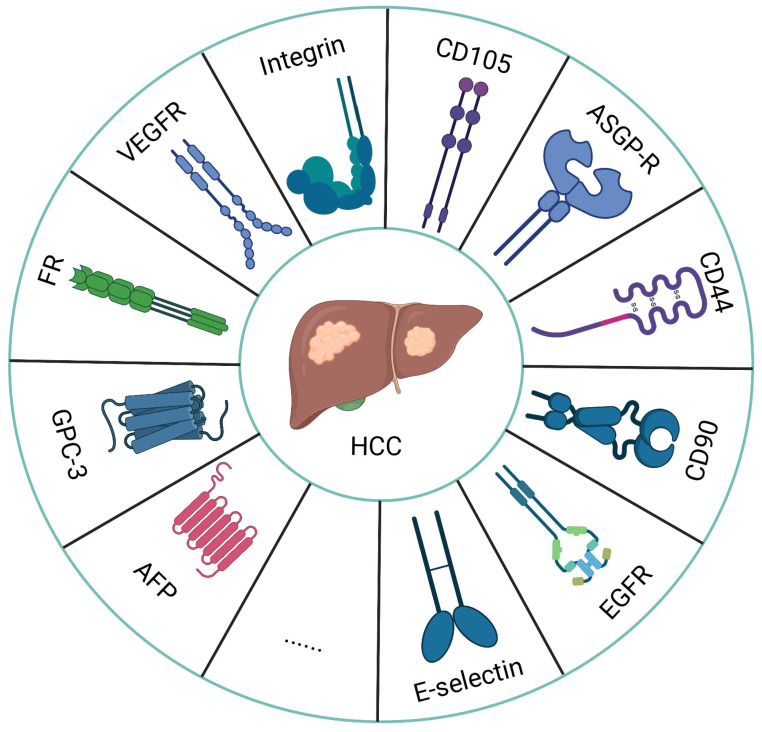
The above is a list of the receptors that are overexpressed in HCC cells.

As is well known, HCC is a highly heterogeneous tumor [8], and identifying specific receptors that are overexpressed in the process of tumor development is particularly important for its early detection and treatment. At the same time, personalized medicine and cancer imaging with a focus on specific receptors have emerged as a major trend in cancer therapy and imaging [28]. For the molecular imaging of HCC, the specificity and sensitivity of the probe are largely determined by the selected targets. With the development of research on the tumor microenvironment of HCC, more and more molecular targets have been explored for molecular imaging (Figure 2) [40,41]. For example, alpha-fetoprotein (AFP), Glypican-3 (GPC3), and folate receptor (FR) can be found on the tumor cell surface, but are rarely or not expressed in healthy cells. With an increase in the volume of the tumor and an increase in the demand for nutrients and blood supply, to meet the needs of growth, the expression of tumor angiogenesis receptors such as the vascular endothelial growth factor, vascular endothelial growth factor receptor (VEGF/VEGFR) and integrin receptor α_v_β_3_ increase significantly compared to in normal tissue [42]. Hence, specific cell-surface biomarkers that respond to tumorigenesis, progression and metastasis can be used as targets for HCC-targeted imaging. Table 2 lists recent studies from the literature on MRI-based targeted molecular imaging of HCC cell-surface molecular biomarkers.

## 3. The Selection of Ligands

When identifying the targets on tumor cells, in order to achieve the purpose of targeted imaging and treatment, the selection of ligands that can specifically bind to the target is crucial. Table 3 summarized the advantages and disadvantages of common ligand types. Among them, antibody is widely used in molecular imaging because of its high specificity and targeting, which is a traditional targeting method, as described in this review, such as anti-AFP antibodies [43,44,45,46], anti-GPC-3 antibodies [52,53,54,55], and anti-VEGF/VEGFR antibodies [67,68,69,70]. In these studies, the high concentration aggregation of antibody-modified molecular probes in the tumor area and the high uptake rate of cells were demonstrated, and an excellent image quality was obtained in subsequent targeted imaging. Antibodies have exceptional affinity and specificity, and their affinity for corresponding targets is frequently greater than that of the majority of peptides [96,97]. The binding modification of antibodies has a negligible effect on their targeting ability, which is highly favored by researchers [98,99,100]. Thus far, antibody preparation technology has matured and is capable of mass production, making it widely used in the field of biological medicine [101]. However, there are many drawbacks of using the antibody as a ligand. Antibodies have a high molecular weight, and their ability to penetrate tumor tissue is limited [102]. Additionally, the structure of antibodies is complicated, limiting its ability to fold [100]. Moreover, the antibody exhibits immunogenicity and is easily phagocytized nonspecifically by the mononuclear phagocyte system [102]. Because the antibody metabolic cycle is long in vivo, the imaging quality is not always optimal [103]. Last but not least, antibody fabrication is costly.

Another targeting method uses bioactive peptides, one of which is called the phage display library [47,48,49,50,71], which was originally proposed by George Smith in 1985 [104]. This technology has been widely used in many fields, such as oncology, cell biology, drug delivery, etc. In terms of cancer-targeting imaging and drug delivery, a variety of membrane proteins on the surface of cancer cells can be used as potential targets. Phage-display technology can screen bioactive peptides that can bind to the corresponding receptors, such as TJ12P1 and L5-peptide-targeting GPC-3 [47,50], and bioactive peptides targeting integrin, with the sequence of CRWYDDENAC (RWY) [71]. In addition to the use of phage-display technology, high affinity peptides can also be obtained through natural screening, such as the arginine-glycine-aspartic acid (RGD) peptide, which exists in an extracellular matrix, and specifically binds to integrin receptors expressed on cell surfaces to mediate adhesion [102]. These receptors are also involved in the initiation and progression of tumors, so the RGD peptide can be used as one of the targets for early targeting [72,73,74,75,76,77]. Peptide, as a ligand, has a simple composition and low molecular weight and manufacturing cost, and it also offers a high degree of editability and an outstanding self-assembly ability [102]. Compared to antibodies, it possesses more tissue penetration and non-immunogenicity, as well as better stability and rapid blood clearance [100,101]. However, high-specificity peptides must be strictly screened, and the workload associated with them is large. More importantly, the optimal peptide cannot be screened out every time.

In addition to the abovementioned methods that use antibodies and peptides as ligands, aptamers have also been developed and applied in the synthesis of specific ligands. Aptamers can be understood as a specific binding target with a three-dimensional structure that can be folded according to a clear definition, usually exhibiting a single-stranded DNA or RNA sequence [99]. Various interactions such as van der Waals forces, hydrogen bonding and electrostatic attraction confer ideal affinity and specificity to the aptamer. It is small in size, so it has good tissue permeability. Because aptamers have a strong recombination ability, theoretically, they can screen any given target, and overcome the limitation of cell lines and animal needs, and achieve non-toxicity and non-immunogenicity [99]. Although their appropriate screening is time consuming, laborious and this process often requires more funds and resources, aptamer synthesis can be mass produced, robust and cheap once a suitable aptamer is successfully determined, which is a great advantage compared to the use of antibodies. Moreover, researchers have successfully screened many aptamers for relevant biomarkers, and studies in this review using aptamers for targeted imaging and therapy have all achieved ideal targeting effects [51,79,80,81]. Similar to the antibody, an aptamer can reduce the impact of binding modifications on the targeting performance, but it has better stability than the antibody [98]. However, the use of aptamers also has shortcomings, for instance, although some aptamers have demonstrated excellent performance during in vitro experiments, their targeting ability in vivo is not ideal; when injected into the body, they may be affected by non-specific serum binding proteins, leading to a reduced binding efficiency [105].

In addition, special ligands, such as folic acid combined with folic acid receptor and sialic acid combined with asialoglycoprotein receptor (ASGP-R) [57,83], can be used and are collectively referred to as small molecule ligands. The most prominent feature of these molecules is that they are easy to obtain, low cost, and their safety has been verified [27]. However, sometimes, their metabolism in the body occurs too rapidly to meet the needs of delayed imaging [103]. Another problem is that they can lead to off-target effects [26]. For example, folic acid receptors are overexpressed in some rapidly growing healthy cells, such as endothelial cells, which can cause nonspecific targeting, resulting in reduced therapeutic efficacy and enhanced side effects.

## 4. Receptor Targeted Molecular Imaging Probes Based on MRI for HCC Theranostics

### 4.1. Alpha-Fetoprotein

**Figure 3:**
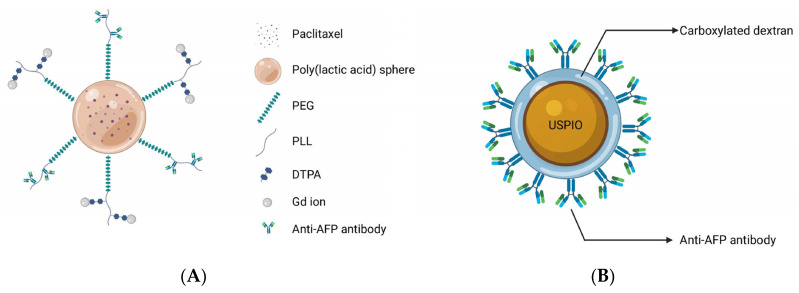
(**A**) The Gd ions used for MR imaging and the antibodies targeting AFP were connected to the outer surface of spherical micelles, and the paclitaxel were encapsulated in the core of micelles for treatment to achieve the purpose of theranostics; (**B**) Carboxylated dextran was used to modify the surface of USPIO directly. A single particle surface has a high coupling efficiency and can couple 12 anti-AFP ant.

As one of the earliest tumor markers to be found, alpha-fetoprotein (AFP) has been routinely employed in a variety of HCC surveillance and detection procedures for decades [8]. Since 1964, Tatarinov et al. found a strong correlation between a high concentration of AFP and the diagnosis of HCC and further investigations demonstrated that AFP is a sensitive marker for HCC diagnosis, its efficacy evaluation, and prognosis [106,107]. AFPs can be used as molecular targets for improving diagnostic and therapeutic efficacy owing to its significant overexpression in HCC tumor cells compared to normal tissues. Liu and co-workers demonstrated the theranostic effects of polymeric micelles with the surface-linked biotinylated AFP antibodies with a biotin–avidin reaction in tumor-bearing Kunming mice after intravenous administration, and discovered that polymeric micelles demonstrated significantly higher signal intensity and a longer imaging duration in the tumor, owing to the specificity of AFP targeting effects and Gd-ion chelation with the micelles (Figure 3A) [43]. Additionally, tumors in the treated animals were greatly suppressed following therapy. Because AFP antibodies enhance the cellular uptake of micelles, chemotherapeutic agents encapsulated in the core of micelles are released to kill tumor cells. Li et al. [45] made a probe using anti-AFP antibodies, which was conjugated with carboxylated dextran-coated USPIONs. Each of the USPIOs can bind twelve antibodies, and this nanomaterial was highly efficient in HCC detection (Figure 3B). In addition, in their study of multimodal imaging, Chen and co-workers proved the feasibility of MRI/fluorescence dual-mode imaging by using AFP-modified fluorescent magnetic probes [46].

**Figure 4:**
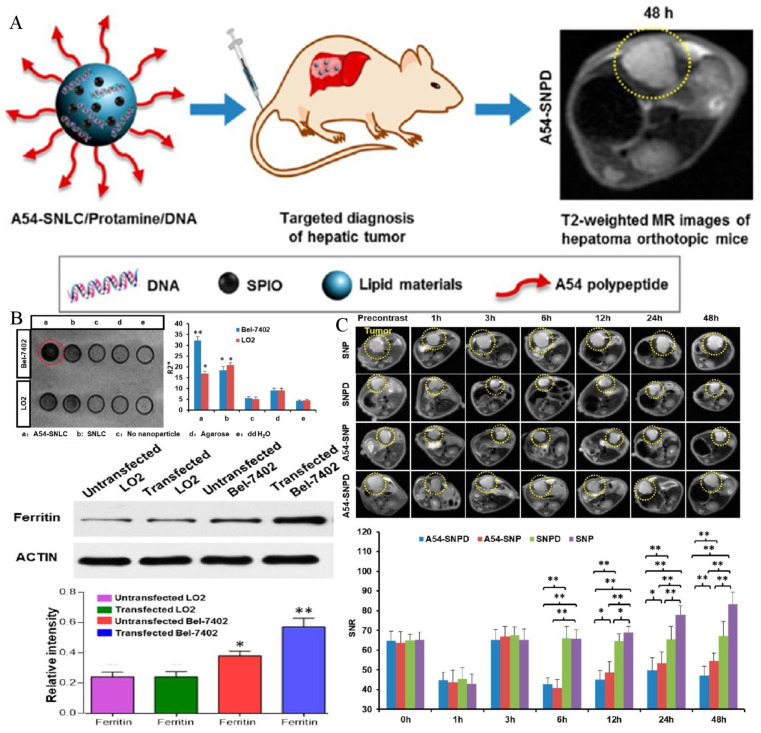
(**A**) A scheme to illustrate the structure of active dual-target nanostructured lipid carrier and the targeted imaging effect in HCC orthotopic mice. (**B**) Cells can specifically uptake the lipid carrier and increase the expression of intracellular ferritin. (**, *p* < 0.01, *, *p* < 0.05, compared with control group). (**C**) T2-weighted imaging of HCC orthotopic mice in vivo (**, *p* < 0.01, *, *p* < 0.05). Reproduced with permission Ref. [109]. Copyright 2017 American Chemical Society.

Since AFP is widely expressed in HCC cells, gene imaging and treatments based on the AFP promoter are also reported in the literature. The ferritin gene, which is a reporter gene, was ligated with AFP promoter to construct the plasmid, which was delivered to the local area by a nanodrug delivery system. Hence, transferrin receptors were highly expressed in transfected HCC cells, resulting in intracellular iron deposition, which was conducive to the early detection of MRI [108,109,110]. Zhang and co-workers demonstrated that the ferritin gene was ligated with the AFP promoter for intracellular transfection by non-viral vectors for the first time. With these endogenous contrast agents, carried by a vector polyethyleneimine-β-cyclodextrin with high safety and stability, gene imaging was successfully achieved [108]. For MR images, the increased intracellular iron accumulation resulted in signal alterations in the lesion region. However, this trial was limited to the cell experiment, and the plasmid carrying the AFP promoter and ferritin gene was introduced into the cells only by transfection to achieve gene imaging. The actual imaging level in living animals remains to be further studied. In another study conducted by Lu, the endogenous imaging of the ferritin reporter gene was successfully verified in the orthotopic HCC model of living animals [109]. Researchers have constructed a nanostructured lipid vector, whose surface is modified by the A54 peptide, and the peptide can be specifically recognized and absorbed by the surface receptors of Bel-7402 cells. The lipid vector contained SPIONs and plasmids containing AFP promoter and ferritin reporter genes. Under the dual synergistic effects, the T2 value of the tumor was successfully reduced, and the sensitivity and specificity of HCC diagnosis were greatly improved (Figure 4).

In summary, AFP has been acknowledged in the diagnosis of HCC as the first and most extensively utilized tumor marker of HCC. Molecular imaging based on AFP also has significant promise, and when combined with several new carriers, it demonstrates excellent targeting for HCC lesions. Additionally, due to its widespread expression in HCC tissues, gene-level imaging and therapy based on the AFP promoter have also shown promising outcomes. However, while recent scientific advances are noteworthy and of interest, the sensitivity of targeting AFP still requires improvement [108]. As a result, additional research should be conducted to fill this gap.

### 4.2. Glypican-3

Glypican-3 (GPC-3) is a type of biomarker for HCC that has drawn attention because of its unique biochemistry and targeted properties [111,112,113,114,115]. GPC-3 is a membrane proteoglycan that is connected to the cell surface by a glycosylphosphatidylinositol anchor and belongs to the heparan sulphate proteoglycan family [116,117]. In recent years, GPC-3 has become a popular research topic due to its characteristics. GPC-3-based molecular imaging and treatment may be an efficient and potentially valuable method for treating HCC.

**Figure 5:**
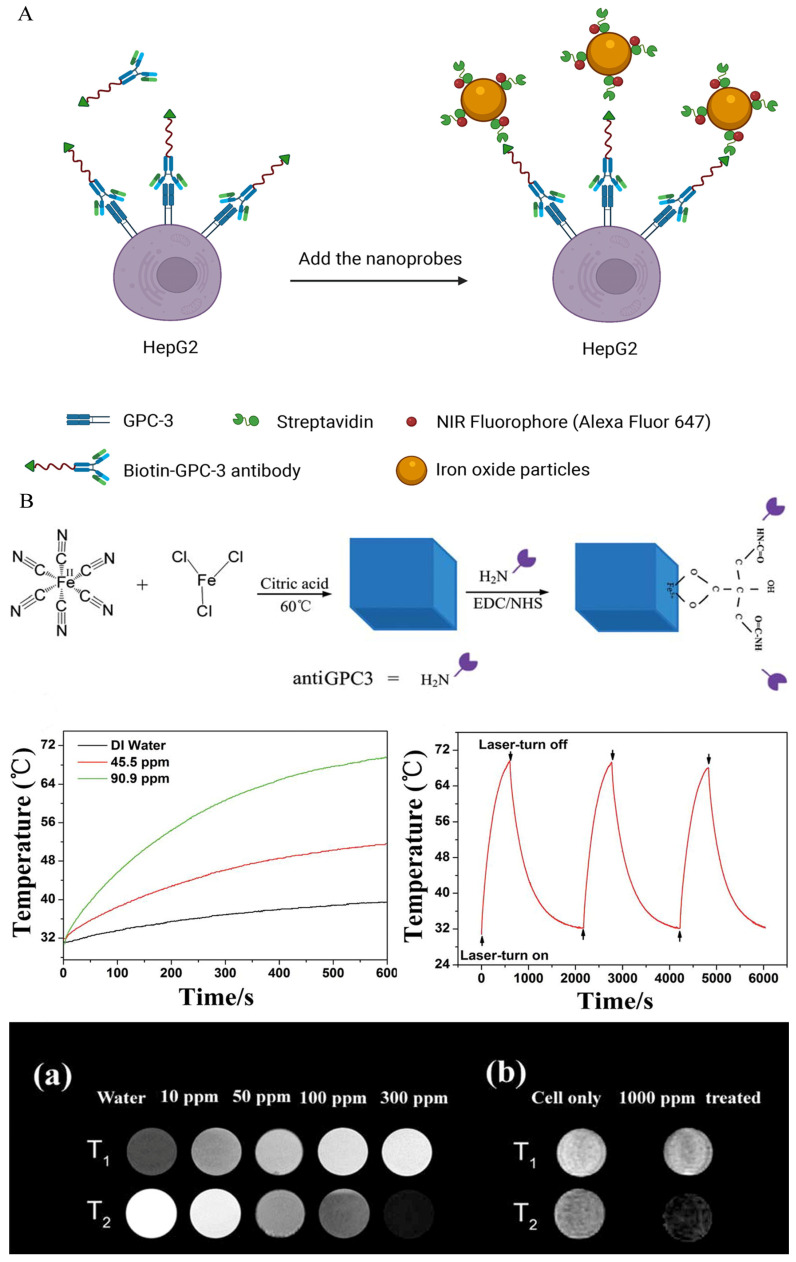
(**A**) Two-step method achieved MRI/NIRF dual-modal imaging [54]. HepG2 cells were incubated with the biotin-conjugated GPC-3 antibodies, and then incubated with dual-modal nanoprobes carrying streptavidin. (**B**) Anti-GPC-3 PBNPs for targeted MR imaging and photothermal ablation in vitro. (**a**,**b**) exhibited images of different concentrations of anti-GPC-3 PBNPs under T_1_ and T_2_ weighted imaging, and MR imaging after incubated with tumor cells, respectively. Reproduced with permission Ref. [55]. Copyright 2014 Royal Society of Chemistry.

**Figure 6:**
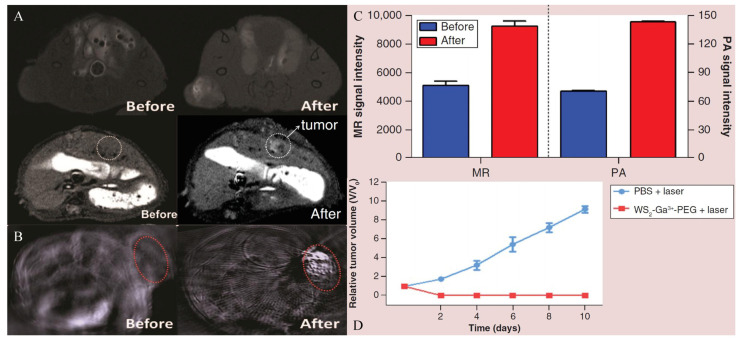
WS_2_-Ga^3+^-PEG-peptide nanoprobe served as a bimodal imaging and photothermal agent. Reproduced with permission Ref. [47]. Copyright 2018 Future Medicine LTD. (**A**) The MR imaging in subcutaneous and orthotopic HCC models. (**B**) The photoacoustic imaging in subcutaneous HCC models. (**C**) The quantified signal intensities of MR and photoacoustic imaging. (**D**) Effect of photothermal therapy in subcutaneous HCC models.

**Figure 7:**
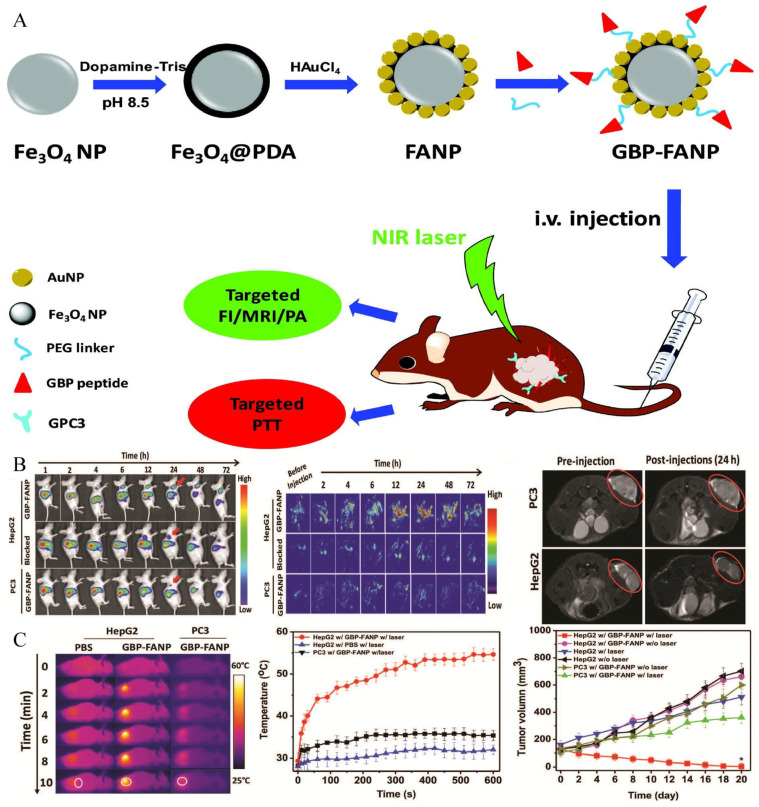
The synthesis and theranostic applications of GBP-FANP. Reproduced with permission Ref. [49]. Copyright 2019 Royal Society of Chemistry. (**A**) The synthesis of GBP-FANP and design of trial. (**B**) Non-invasively targeted MR imaging, fluorescence imaging and photoacoustic imaging in vivo. (**C**) Photothermal treatment effect of GBP-FANP.

The exploration of GPC-3 for theranostic applications of HCC began in 1997. Hsu and co-workers described, for the first time, that GPC-3 mRNA was overexpressed in 74.8% of HCC tissues and proved its potential imaging and therapeutic value [118]. In 2001, Zhu et al. published similar findings, stating that the expression of GPC-3 mRNA was elevated in 83% of HCC tissues compared to nodules, liver cirrhosis tissues, and normal liver parenchyma [119]. Since then, researchers have directed their efforts to the prospective uses of GPC-3. GPC-3 monoclonal antibody, as a solution for targeted imaging, has been used in recent studies [54,55], and it can target the GPC-3 receptor expressed on HCC cell surfaces. James et al. reported the targeting ability of GPC-3 monoclonal antibody on GPC-3-expressing cells. Firstly, HepG2 cells with a high expression of GPC-3 were incubated with the biotin-conjugated GPC-3 antibodies. Then, streptavidin and NIR fluorophore were ligated on the surface-modified iron oxide nanoparticles, and streptavidin was successfully combined with the biotin on the GPC-3 antibody. Finally, the synthesized probes were incubated with cells, and dual modal imaging of near-infrared fluorescence and MR imaging were successfully achieved (Figure 5A) [54]. Li and co-workers illustrated that GPC-3 antibody-functionalized PBNPs (a prototype of mixed-valence transition metal hexacyanoferrates) displayed good capabilities in both targeted MR imaging and photothermal treatment (Figure 5B) [55,120]. However, despite its strong affinity for GPC-3, the antibody’s large size may have adverse consequences, including inadequate imaging pharmacokinetics, poor tumor penetration, and higher immunogenicity [121,122]. Peptides may be another option for addressing these issues. Highly sensitive and specific peptides targeting GPC-3 can be screened using the phage-display peptide library [123]. Minimal molecular weight, simplicity of customization, and low scale-up costs are all benefits of peptide-based probes [124]. For example, by combining the strong paramagnetic properties of gadolinium ions with the excellent near infrared absorption properties of WS_2_, Song et al. synthesized an MRI and photoacoustic-imaging bimodal nanoprobe to provide an effective targeting specificity of tumor cells [47]. The generated nanoprobes were shown to be compatible with the physiological environment and to have no detectable toxicity both in vitro and in vivo, and outstanding imaging effects were observed in MRI and photoacoustic imaging. Furthermore, dual-modal imaging determined the macroscopic outline of the tumor and improved the effect of NIR-induced tumor ablation (Figure 6). Similarly, ligands screened using phage-display peptide library technology also showed excellent targeting and affinity [49]. Tian et al. modified the GPC-3 binding peptide (GBP) identified by phage display technology on the surface of a traditional Fe_3_O_4_ Core/Au shell nanocomplex (FANP), in which the photothermal effect was mediated by the Au shell and the MR imaging was mediated by Fe_3_O_4_ nanoparticles (Figure 7) [49]. The experimental results showed that after intravenous administration, the GBP-FANP increased gradually in tumors. After 24 h, imaging showed that the local aggregation of GBP-FANP reached the peak and photothermal therapy was performed by laser. Compared with GPC-3-negative tumors, GPC-3-positive mice tumors were significantly inhibited, and no systemic toxicity was found, which proved the feasibility of this imaging method and treatment strategy.

According to previous research, GPC-3 has a greater sensitivity and specificity, as well as a higher expression level than AFP [125,126,127], with approximately 70% of HCCs detected with the high expression of GPC-3 [115]. Although AFP is widely used in clinical practice, there is still a high false-negative rate. Compared with AFP, the expression rate of GPC-3 in early HCC is significantly higher, especially in patients with tumor diameters of less than 3 cm [118]. Even in AFP-negative HCC patients, GPC-3 still can be detected with high expression [128]. Hence, due to its excellent specificity and sensitivity at early stage HCC, GPC-3 can be used to differentiate between benign and malignant hepatic nodules and in the early diagnosis of small lesions. He and co-workers proved for the first time the feasibility of the diagnosis and treatment of micro-HCC [52]. Their group created a unique organic–inorganic composite nanoprobe that was capable of dual-modal imaging (MRI/NIR-II) and non-invasive photothermal treatment. Deng et al. successfully synthesized a targeted probe FeSe_2_-PEG-Peptide coupled to GPC-3 for contrast-enhanced MRI and photoacoustic imaging. Nanoprobes can accurately judge a nodule whether it is HCC or liver cirrhosis [48]. In addition, since this material has excellent photothermal effects, photothermal treatment can be performed after targeted imaging.

GPC-3 can also be employed for high-efficiency-medication delivery due to its high expression rate. For instance, SPIONs and sorafenib were encapsulated in polymer micelles, which could trigger drug release through an intracellular reduction reaction and a change in the pH value. This dual-trigger mechanism ensured the precise release of drugs in situ [53]. To be more precise, endocytosis mediated by the GPC-3 receptor stimulates the entry of micelles into cells, and subsequent intracellular glutathione reduction reactions and changes in the pH value enhance the release of SPIONs and sorafenib from micelles. Furthermore, the release of SPIONs assists in noninvasive tumor identification and in the monitoring of in vivo drug administration by MRI.

Compared with simply using GPC-3 or AFP targets separately, dual-targeted imaging may enhance the early detection rate of HCC. Ma et.al successfully detected early stage malignant nodules with dual-targeted imaging [44]. In contrast to targeting AFP alone on the HCC cell surface, Ma et.al proposed that an AFP/GPC-3 double antibody-labeled probe which can target AFP and GPC-3 simultaneously could potentially increase the detection rate of HCC, and improve the efficacy in detecting heterogeneous micro malignant HCC tumors. In their study, the properties of targeting and the sensitivity of the dual-labeled probe were higher than single-labeled probes.

In summary, GPC-3 has attracted great interest in recent years due to its unique biochemical and targeted properties. The high expression of GPC-3 in tumor tissues makes it an ideal target for the imaging and treatment of HCC. Especially, in the diagnosis of tiny malignant nodules, GPC-3 showed higher sensitivity and specificity than AFP, and the experiments explored in this chapter also achieved ideal results. In terms of the corresponding ligands of GPC-3, researchers have designed a variety of ligands for selection such as antibodies, peptides and aptamers. Therefore, GPC-3 may be a molecular target with great potential in the future.

### 4.3. Folate Receptors

Folic acid is a vitamin that is required for cell growth. Folate receptors are primarily responsible for transporting folic acid within mammalian cells and tissues. Under physiologic conditions, the expression level of receptors in normal cells is relatively conservative [65]. Nevertheless, in malignant tumor tissues, rapid cell division increases the demand for folic acid, and the corresponding expression of receptors is significantly increased [129]. Thus, the surface modification of nanoparticles with folic acid may indicate significant potential for the development of a novel strategy with which to improve the efficiency of cancer diagnosis and treatment [130,131].

Compared with other ligands such as antibody-based targeting moieties, folic acid has a number of potential advantages as a targeting moiety, including ease of synthesis, low molecular weight, strong receptor affinity in tumor tissue, and outstanding stability and biocompatibility [132]. Hence, Folic acid has been covalently conjugated to anticancer drugs, dendrimers, polymers, and metallic compounds with modified surfaces for the detection and therapy of HCC [133]. For example, a folic-acid-functionalized gadolinium-loaded nanodroplet was synthesized as a dual-modal MRI/ultrasound contrast agent to target HCC cells [58]. The nanodroplets showed increased cellular absorption and selective accumulation in the tumor location, intensifying the MRI signal for tumor areas with a high r1 relaxation, which is even greater than the relaxation of the currently available clinical contrast agent (Gadovist). In another study, a folate-modified ultra-small magneto-gold nanoparticle was synthesized. This nanoparticle not only increased the imaging mode (quad-modality imaging), but also combined photothermal treatments for in situ tumors [63]. In vivo therapeutic experiments showed that folate-modified ultra-small magneto-gold nanoparticles exhibited high photothermal antitumor efficacy and reduced the tumor size compared with the control group. There are many similar experimental studies available about the targeting of folate receptors. The studies introduce the application of molecular imaging probes based on folic-acid surface modification in the diagnosis and treatment of HCC using the following four aspects: Targeted delivery of siRNA, pH-sensitive release, folate acid-modified polymer-based nanoparticles and folate acid-modified metal particles.

**Figure 8:**
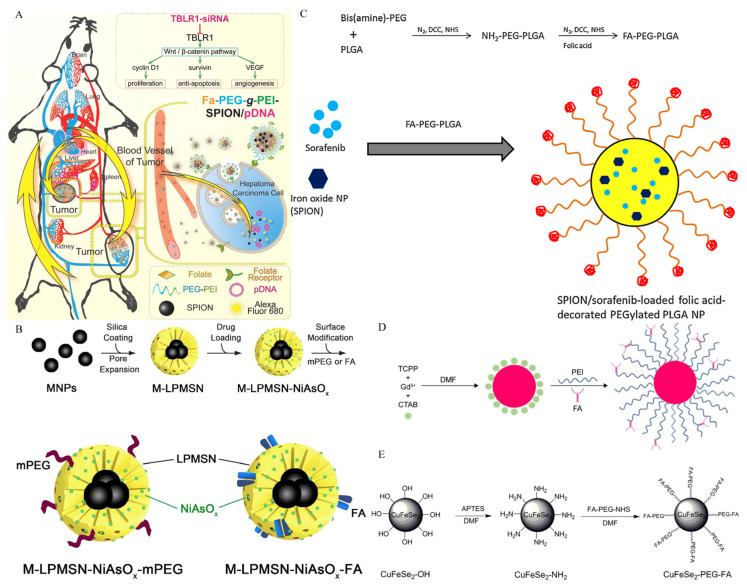
Different types of molecular imaging probes targeting folate receptors. (**A**) A schematic diagram of tumor-specific uptake and in vivo distribution of nanocomplexes carrying siRNA and SPIONs for treatment and diagnosis. Reproduced with permission Ref. [56]. Copyright 2015 Hepatology. (**B**) The synthesis of pH-sensitive magnetic macro-porous mesoporous silica nanoparticles. Reproduced with permission Ref. [60]. Copyright 2019 IOP Publishing. (**C**) Synthesis of folic acid conjugated PEG-PLGA diblock polymer. SPION and sorafenib were co-encapsulated into folic acid-PEG-PLGA to produce multifunctional nanoparticles. Reproduced with permission Ref. [61]. Copyright 2015 Elsevier. (**D**) Synthesis of folic acid-targeted metal organic frameworks. Reproduced with permission Ref. [57]. Copyright 2019 Dovepress. (**E**) Folic acid-targeted CuFeSe_2_ nanoparticles that can be used in MRI/CT dual-modal imaging. Reproduced with permission Ref. [65]. Copyright 2021 Dovepress.

Researchers have discovered that transducing β-like protein 1-related protein (TBLR1) is a major HCC oncogene that is involved in the anti-apoptosis, proliferation and angiogenesis of HCC [134]. Guo at al. synthesized a plasmid nanocarrier containing siRNA corresponding to the TBLR1 gene for targeted delivery into HCC cells (Figure 8A) [56]. In the study, TBLR1-plasmid-loaded nanoparticles were prepared, and highly efficient cell endocytosis and excellent in vivo imaging performance were reported. Furthermore, the levels of TBLR1 mRNA and protein in the tumor tissues were considerably lower than in the control group. Wu and co-workers produced a novel type of folate-functionalized, SPIO-loaded nanoparticles to transport survivin-siRNA (surviving, which plays a key role in oncogenesis) to HCC cells [62]. These results revealed a silencing effect and superior MR imaging in vitro.

The targeting delivery of therapeutic drugs mediated by pH-sensitive release has also been reported in folic acid-based nanoparticles. For example, tumor pH-sensitive nanoformulated triptolide coated with folic acid for targeted drug delivery [59]. The developed nanomedicine’s physicochemical properties indicated that it was suitable for drug delivery applications due to its pH-dependent release. Additionally, in orthotopic mouse models, targeted drug delivery significantly reduced the tumor burden and improved survival without toxicity. However, combined imaging may be a better method to achieve theranostic effects than targeting drug release alone. Chi and colleagues effectively loaded the precursor medication, arsenic trioxide, into the pores of porous mesoporous silica nanoparticles, which include magnetic iron oxide particles for MR imaging in their core and generated the targeted nanoparticles. The surface modification of the as-obtained nanoparticle was accomplished by linking folic acid in order to achieve the dual function of imaging and targeted drug administration simultaneously (Figure 8B) [60]. The releasing curves demonstrate arsenite’s sensitive discharge in an acidic environment. Experiments in vivo with tumor-bearing mice revealed enhanced anticancer activity and exceptional imaging capability.

Folic-acid-modified polymers as imaging/drug carriers have been reported in the diagnosis and treatment of HCC. For example, Li et al. successfully developed a novel PLGA-based composite nanoparticle, which encapsulated sorafenib and SPIONs together and coupled folic acid onto the surface of nanoparticles (Figure 8C) [61]. In vitro cell experiments revealed that, as compared with the control group, the cellular uptake of nanoparticles was enhanced, and the proliferation of tumor cells was effectively inhibited by sorafenib. Additionally, enhanced MRI properties have also been reported. Similarly, another study reported that polydopamine-coated magnetite nanoparticles with folic-acid targeting groups had a good killing efficiency when used in combination with the chemical and photothermal therapy of HCC cells, and the magnetic particles also performed well on MR imaging [64].

In addition to some traditional paramagnetic materials such as iron oxide, other metal particles can also be used as diagnostic and therapeutic agents for targeted folic receptors. Gadolinium-porphyrin metal–organic frameworks with excellent MR imaging and fluorescence imaging capabilities were synthesized (Figure 8D) [57]. Dual-modality imaging was achieved by targeting tumor tissue with folic acid conjugated. In addition, as it is a photosensitizer, good photodynamic therapy effect was reported in the research. In another study, a folic acid-targeted CuFeSe_2_ nano-contrast agent was fabricated and exhibited excellent imaging performance and targeted capability in MRI/CT dual-modality imaging in vitro and in vivo (Figure 8E) [65].

Folic acid has distinct features, such as high receptor affinity, low molecular weight and excellent biocompatibility, making it a promising target for biomedical applications. Similarly to other targeted molecules, the most direct advantage of folic acid is its ability to act as a targeted molecule in the drug-delivery process. In addition, folic acid has good biocompatibility and accessibility, showing great potential in molecular imaging. Much progress has been made in combining folic acid with carrier drugs and imaging agents to maximize their anticancer and diagnostic effects.

### 4.4. VEGF/VEGFR

**Figure 9:**
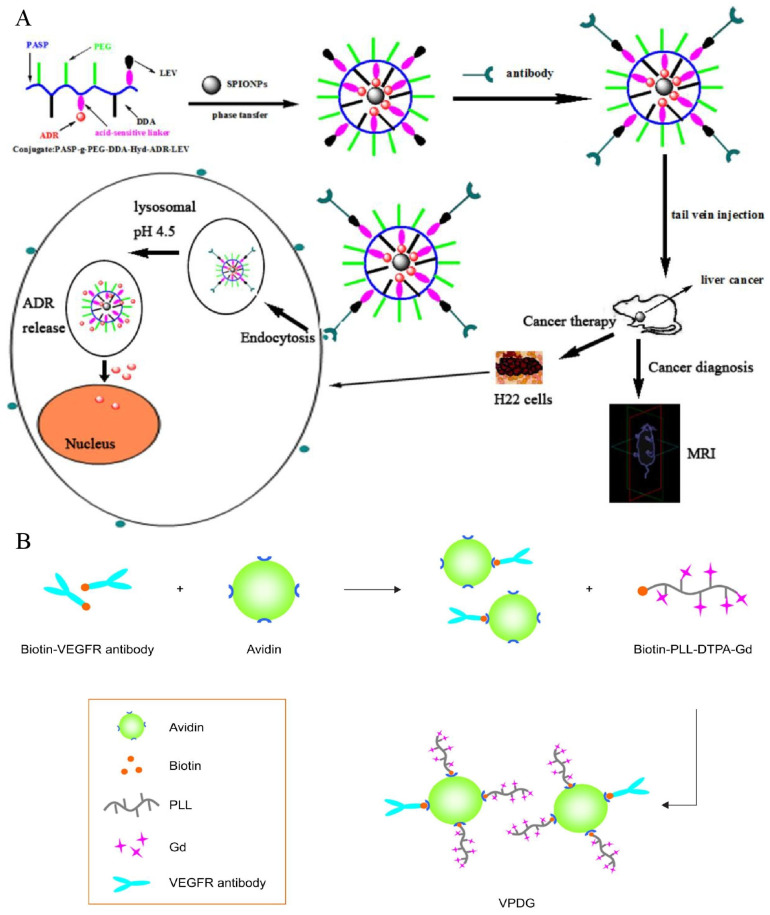
(**A**) Preparation of molecular probes targeting VEGF. Reproduced with permission Ref. [68]. Copyright 2013 Wiley Online Library. (**B**) The synthesis of probes targeting VEGFR. Reproduced with permission Ref. [69]. Copyright 2017 Dovepress.

VEGF is strongly expressed in tumor tissues and its expression has been found to be proportional to the degree of malignancy for tumors [42,135]. Additionally, the corresponding receptor is VEGFR, which is highly expressed in the majority of tumor cells as well as endothelial cells involved in tumor neovascularization [136]. The binding of receptors and ligands to activate downstream signals plays a crucial function in tumor angiogenesis, tumor tissue development, and invasion [137]. Hence, when building targeted probes, there are two options for targeting VEGF or VEGFR. One of the methods is targeting VEGF (Figure 9A). For example, studies on probes targeting VEGF have been reported [67,68]. Liu et al. used the anti-VEGF antibody to modify polymeric particles containing gadolinium and Huang et al. synthesized the MRI-visible and VEGF targeted drug delivery system, both of which have shown the feasibility of targeting VEGF. All of them exhibited the ability to efficiently target HCC cells for early detection.

Similarly, as previously reported, VEGFR overexpresses in tumors and neovascularization [138]. Therefore, targeting VEGFR may be another promising strategy (Figure 9B) [139]. A specific magnetic imaging probe, based on PLL, and a connected paramagnetic substance, gadolinium with a DTPA chemical bond, when targeting VEGFR, successfully diagnosed HCC in the early stage [69]. A biotin–avidin reaction was used to bind VEGFR-antibodies to PLL. No obvious cytotoxicity was found in vitro, and the nanoparticles significantly increased the internalization rate of VEGFR-positive HepG2 cells. The tumor signal intensity and time of duration significantly increased in H22 mice subcutaneous tumor models. Similarly, Liu et al. synthesized a multifunctional pH-sensitive nanoparticle for the diagnosis and treatment of HCC [70]. Compared with the above synthesis process, Liu introduced pH-sensitive materials, of which gadolinium ions were connected to the external DTPA residue, and sorafenib was wrapped in nanoparticles for treatment. Drug release was achieved under the acidic condition of the tumor microenvironment. At a pH of 5.0, in vitro testing revealed that medication release might approach 99%, and for in vivo antitumor studies, compared with oral or intravenous sorafenib, these pH-sensitive materials have more obvious antitumor effects in mice bearing H22 tumors. It exhibited an improved resolution and a longer imaging period (more than 90 min) when used as a contrast agent in the diagnosis of tumor-bearing mice.

### 4.5. Integrin

Integrin is comprised of the following two subunits: the alpha and beta subunits. These heterodimeric cell surface receptors are strongly associated with malignant biological characteristics such as tumor angiogenesis, invasion, and metastasis through the mediation of cell adhesion and signal transmission [140,141,142]. Integrins are extensively expressed on neovascular endothelial cells and HCC tumor cells, whereas they are rarely found on the surface of normal hepatocytes [143,144]. As a result, integrin may be an appropriate target for the early stage detection and therapy of HCC. Integrin is a significant component of the cell-adhesion molecule family, consisting of several subtypes, and at present, the main integrin molecules used in the study of magnetic molecular imaging probes are integrin α6 and integrin α_v_β_3_ [71,72,73,74,75,76,77,78,145].

**Figure 10:**
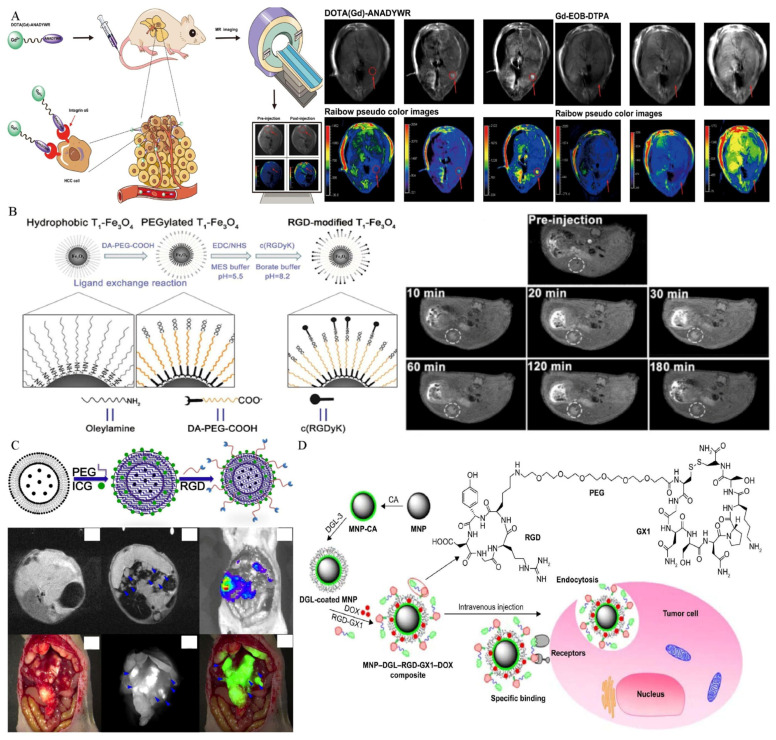
Research about various types of probes targeting Integrin. (**A**) Integrin α6 targeted MR molecular imaging probe for HCC detection. Reproduced with permission Ref. [71]. Copyright 2021 Dovepress. (**B**) The synthesis of integrin α_v_β_3_ active targeted T1 molecular imaging probe and MR images. Reproduced with permission Ref. [73]. Copyright 2016 Dovepress. (**C**) MR/NIRF dual modal imaging probe. Reproduced with permission Ref. [78]. Copyright 2017 Dovepress. (**D**) Schematic diagram of doxorubicin-loaded molecular imaging probes. Reproduced with permission Ref. [76]. Copyright 2017 Dovepress.

Integrin α6 subunit can form integrin α6β1 subtype and α6β4 subtype with integrin β1 subunit or β4 subunit heterodimer. Most of them bind to extracellular matrix laminin and mediate adhesion between cells and between cells and the extracellular matrix [146,147,148]. Integrin α6 expression was shown to be considerably greater in early stage HCC tissues than in surrounding normal tissues [149,150], and was related to a worse prognosis and malignancy in previous investigations [151,152]. Hence, the overexpression of integrin α6 in early HCC has an extraordinarily high positive rate, making it a possible diagnostic biomarker for early HCC detection [71,145]. For example, Lin et al. obtained a peptide with a high affinity to integrin α6 by alanine scanning and linked it to Gd ions. An optimised MR probe, specific for integrin α6, was produced, which can detect small nodules (approximately 1 mm) in mice (Figure 10A) [71], indicating the possibility of using this integrin α6-targeted MR probe to identify HCC, particularly tiny malignant nodules.

Integrin α_v_β_3_ is another commonly used imaging target and is composed of α_v_ subunit and β_3_ subunit. The extracellular region of the α chain from integrin α_v_β_3_ can specifically recognize arginine-glycine-aspartic acid (RGD) polypeptides. Moreover, integrin α_v_β_3_ is highly expressed in various tumors, including HCC cells and the neovascular endothelial cells of tumors [153]. Hence, molecular imaging probes can include the RGD polypeptide, which has the potential for use as a target-imaging agent for HCC due to the high affinity of RGD polypeptides for integrin receptors [75]. Active-target T1 imaging of HCC tumors as small as 2.0 mm was carried out for the first time by Jia et al. [73]. A 2.0 mm tumor can be detected using RGD-modified Fe_3_O_4_ with T1 contrast enhancement (Figure 10B). Before this trial, silica-coated superparamagnetic iron oxide core–shell nanoparticles connected with paramagnetic gadolinium complex and RGD peptide as ligands were successfully synthesized, and T1 and T2 weighted dual-modal imaging was achieved [77]. Chen et al. developed a novel dual-mode probe based on MR and NIRF imaging and demonstrated its viability in a nude mouse HCC model. The results indicated that it can likely improve the accuracy of liver-tumor identification and guiding during resection (Figure 10C) [78]. In the targeting delivery of siRNA or drugs, promising results were also reported. The use of RGD-modified polyethylene glycol-grafted polyethylenimine functionalized with SPIONs as a carrier for survivin siRNA administration was investigated. It has the potential to modify gene expression in the treatment of HCC and to identify the tumor in vivo as an effective MRI probe [75]. Shen and co-workers designed a novel dual-targeted nanoprobe loaded with doxorubicin. The as-obtained multi-functional nanoparticles with excellent biocompatibility showed tumor-specific accumulation behaviors and significant antitumor activity (Figure 10D) [76].

In summary, with developments in research, integrins have demonstrated a broad range of potential applications in tumor imaging and medication administration. The molecular targeting of integrins provides new ideas for the detection and treatment of HCCs. As this research is still in its early stage, efforts should be directed towards (1) the improvement of the selection of integrin-based imaging agents and drug carriers, (2) verifying the safety of human application, (3) identifying the optimization of imaging effects, and (4) exploring the pharmacokinetics of clinical drugs.

### 4.6. Endoglin (CD105)

Endoglin, also known as CD105, is an endothelial cell membrane glycoprotein that is highly expressed in the neovascularization of cancer cells including HCC [154,155], which was first discovered in 1990s and initially named as the 44G4 antigen [156,157]. Endoglin is a component of the transforming growth factor beta (TGF-β) receptor complex that plays a critical role in angiogenesis and vascular remodeling [81,158]. It is worth mentioning that growing tumor endothelial cells have higher endoglin expression than resting endothelial cells [155,159,160]. As a result, it might be a good target for imaging and treatment [81,157]. In recent studies, specific aptamers with a high affinity to CD105 were successfully screened by conducting an exponential enrichment analysis [79,80,81].

**Figure 11:**
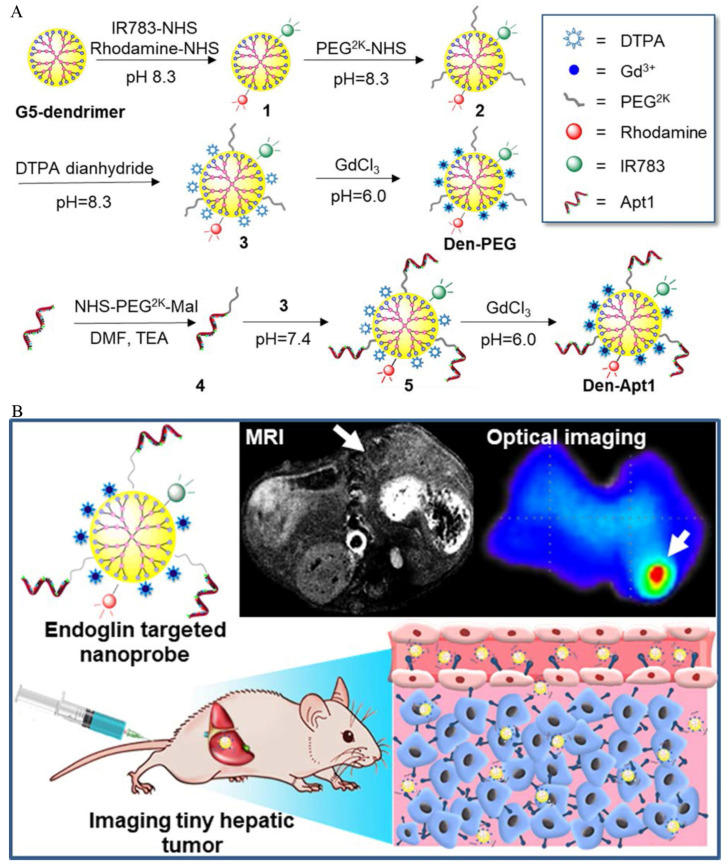
(**A**). Construction of endoglin-targeted MR/optimal dual-mode molecular imaging probe using aptamer as ligand. (**B**). The as-synthesized nanoprobe successfully achieved MR/optimal dual modal imaging in vivo by targeting endoglin overexpressed on the surface of HCC cells and endothelial cells of neovasculature. Reproduced with permission Ref. [80]. Copyright 2018 American Chemical Society.

Based on endothelial glycoprotein targets, Zhou and his colleagues successfully developed a specific MRI/fluorescent imaging aptamer nanoprobe. The dual-mode nanoprobe exhibited MR and fluorescence-imaging capabilities, and was able to perform both MR imaging and fluorescence labelling simultaneously. Unfortunately, this experiment was only limited to the synthesis of probes, the optimization of experimental parameters and characterization, and was not further verified at the cellular and animal levels [79]. In the same year, Yan et al. screened a novel single-stranded DNA oligonucleotide-based aptamer by conducting an exponential enrichment analysis, and conjugated the aptamers to create an MR/optimal dual-targeted nanoprobe that successfully visualized orthotopic HCC tumors that were as small as 1–4 mm in diameter (Figure 11) [80]. Moreover, the dual-modal probe showed excellent accuracy and potential for the edge delineation of invasive HCC and guiding tumor excision. Zhong et al. identified an aptamer that can bind to mouse endoglin molecular (m-END) specifically, by conducting an exponential enrichment analysis. On that basis, they used m-END as the targeted molecular, Fe_3_O_4_ as the magnetic material and prepared the imaging nanoprobes based on carboxymethyl chitosan nanoparticles (mEND-Fe_3_O_4_@CMCS) [81]. In vitro, this probe exhibited excellent biocompatibility and targeting ability, and during an in vivo experiment, local enhancement of the tumor lasted for more than 6 h compared with that before injection in HCC-bearing BALB/c mice.

### 4.7. Asialoglycoprotein Receptor

ASGP-R, a membrane-bound lectin [161], was suggested in a previous study to be implicated in the progression of cancer metastases [162]. ASGP-R expression is abnormally upregulated in HCC, and the receptor can bind to galactose selectively, initiating receptor-mediated endocytosis and facilitating galactose endocytosis into tumor cells [163,164,165]. ASGP-R is therefore considered as a desirable molecular target for theranostic development. By using galactosylated molecules as drug delivery vehicles or imaging media, it is possible to enhance the diagnostic and therapeutic effects of HCC.

**Figure 12:**
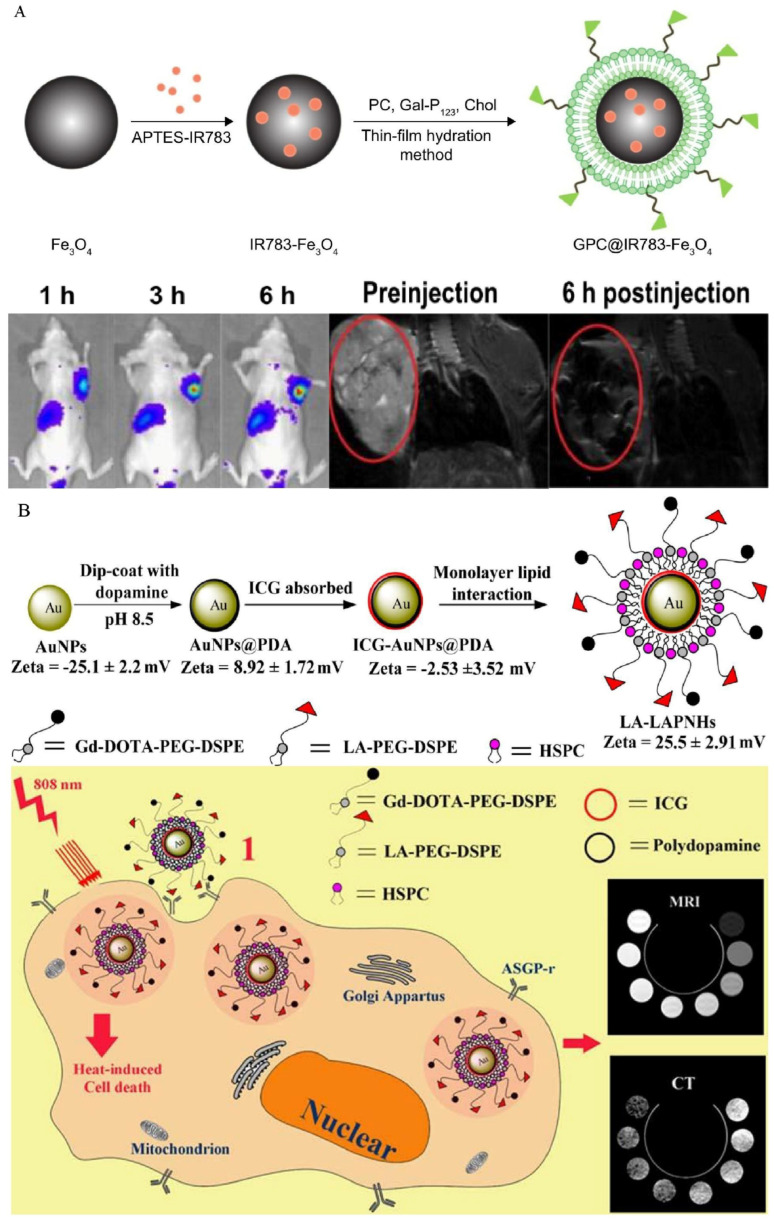
(**A**) The synthesis of MRI/NIRF dual-modal probe and effect of imaging. Reproduced with permission [82]. Copyright 2018, Dovepress. (**B**) The composition of MRI/CT dual-modal probe and theranostic mechanism. Reproduced with permission [83]. Copyright 2014, American Chemical Society.

Liang and colleagues conjugated NIRF to the surface of Fe_3_O_4_ and then modified it with galactose-containing lipids to create dual-mode imaging nanoparticles for HCC cells. By specifically targeting HCC cells overexpressing ASGP-R, the nanoparticles enabled precise imaging. The produced imaging probe exhibits outstanding biocompatibility and MRI/fluorescence performance, indicating that it has significant clinical application potential (Figure 12A) [82]. In another study, dual-modality imaging nanoparticles were prepared using gold nanoparticles for the inner core and loaded this with indocyanine green by coating polydopamine on the surface, and the shell was composed of modified lipids containing gadolinium acid and lactobionic acid that self-assemble on the outer surface. The nanoparticles successfully achieved MRI/CT dual modal imaging and targeted photothermal cytotoxicity. Particles internalized into cells were clearly observed in cell experiments, and the nanoparticles exhibited excellent NIR absorption in the region between 700 and 850 nm, and thus induced significant photothermal cytotoxicity (Figure 12B) [83]. In gene therapy, Cai et al. delivered microRNA-99a into HCC cells by targeting VEGF and ASGP-R targets simultaneously, which successfully inhibited HCC progression [84].

### 4.8. CD44

**Figure 13:**
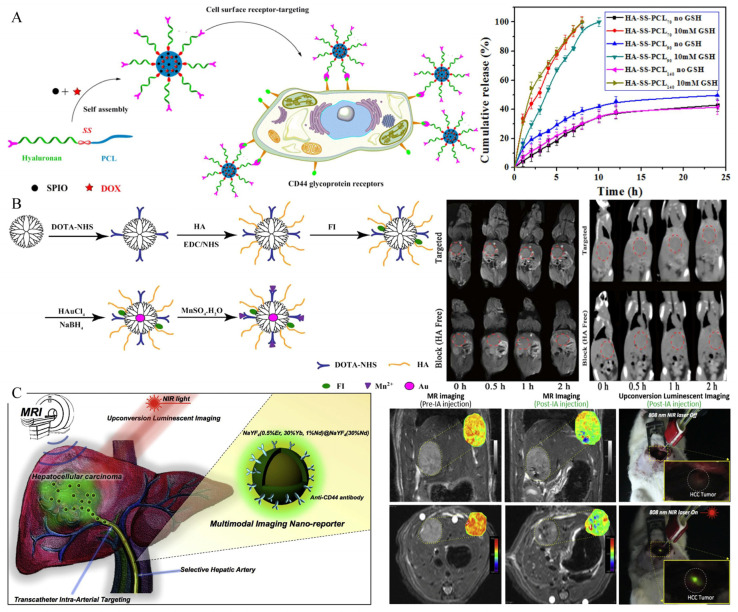
(**A**) Preparation of glutathione-responsive micelles loaded with doxorubicin and SPIONs, and 100% release of doxorubicin in reduction state. Reproduced with permission Ref. [88]. Copyright 2019 Elsevier. (**B**) Preparation and imaging of MRI/CT dual-mode probe. Reproduced with permission Ref. [86]. Copyright 2016 Springer Nature. (**C**) Transarterial infusion of nanoprobes for localization of lesions and determination of surgical margins. This probe can provide MRI and real-time fluorescence imaging for diagnosis and resection. Reproduced with permission Ref. [89]. Copyright 2016 Elsevier.

Cluster Determinant 44 (CD44) is a receptor that mediates endocytosis on the surface of liver-cell membranes [166], but the expression of CD44 receptors on HCC cell membranes can be increased significantly [167,168]. As a primary ligand with a high affinity for CD44, hyaluronic acid is particularly interesting for applications such as the tumor-targeted administration of imaging agents for the detection and treatment of HCC due to its biodegradability, biocompatibility and non-immunogenicity [86,87,88,89,169]. For instance, Yang et al. successfully developed theranostic glutathione-responsive micelles that encapsulated doxorubicin and SPIONs (Figure 13A) [88]. The in vitro drug-release data indicated that the micelles held the potential to release doxorubicin in response to reductant, which was validated by 100% doxorubicin release in the presence of 10 mM glutathione. Through the receptor-mediated mechanism between hyaluronic acid and the CD44 receptor, the micelles loaded with doxorubicin and SPIONs were internalized in HCC cells and exhibited a pronounced antitumor ability and excellent tumor-imaging potential. Wang and coworkers developed multifunctional nanoparticles modified with hyaluronic acid for dual-mode MR/CT imaging of HCC cells overexpressing CD44 receptors (Figure 13B) [86]. The as-obtained nanoparticles exhibited excellent water dispersibility, cytocompatibility, and stability. In both in vitro and in vivo cell experiments, orthotopically transplanted HCC tumors models exhibited satisfactory imaging characteristics.

An invasive but innovative approach to intraoperative transarterial infusion of imaging agents was also reported by Lee et al. (Figure 13C) [89]. The intraarterial infusion of Nd^3+^ doped nanoparticles combined with anti-CD44 monoclonal antibody can achieve MR and the real-time upconversion luminescence imaging of HCC in situ rat models, which is helpful for the intraoperative determination of surgical margins and the detection of small lesions.

### 4.9. Other Types of HCC-Targeted Molecular Imaging Probes

Besides the commonly used targets mentioned above, some other targets are still used in the imaging and treatment of HCC. Liver-cancer stem cells (LCSCs) have recently been considered as a contributor to HCC initiation, relapse and metastasis [170], and CD90 is a key marker for LCSCs. Previous research established a strong correlation between CD90 expression and the malignant nature of HCC [171,172]. Thus, targeting CD90-positive LCSCs for therapeutic or imaging purposes has significant practical implications. Earlier, in 2016, researchers prepared CD90 thermosensitive magnetoliposomes, by targeting CD90^+^ cells in HCC and achieved promising results [91]. However, this scheme has not been combined with diagnosis because they ignored the potential imaging ability of CD90. Chen and colleagues created temperature-sensitive magnetic liposomes in combination with anti-CD90 monoclonal antibodies to target synergistic chemotherapy/magnet hyperthermia and in vivo synchronous imaging. The experimental results indicated that the targeted imaging group’s relative fluorescence intensity was almost double that of the non-targeted group. The targeted group’s T2 relaxation period was substantially shorter than that of the non-targeted group. Compared to single therapy, this theranostic strategy had a considerable anti-tumor impact. In addition, real-time monitoring targeted MR imaging provided clear tumor-imaging and reported therapeutic effects in a timely manner [90].

Epidermal growth factor receptor (EGFR) is a transcription factor which is involved in cell proliferation, metastasis, and angiogenesis [173]. Presently, the aberrant expression of the EGFR and activation of EGFR-mediated downstream signaling pathways have been seen in a large number of human malignant tumors, including HCC. Recently, anti-EGFR targeted imaging and therapy have received a great deal of interest from the medical community [92,93,174]. For example, Chen et al. achieved targeted MR imaging by modifying a peptide with a high affinity to EGFR on USPIO [92]. Specific peptides bind to the EGFR extracellular domain, which is overexpressed on HCC cell surface, thus promoting endocytosis and achieving tumor imaging. Additionally, similar results were obtained in another study targeting EGFR by peptide [93].

On the surface of malignant tumor cells, E-selectin is extensively expressed, and sialic acid may be employed as a particular ligand to attract and bind to the protein [175]. Preliminary research has found that sialic acid functionalized nanomedicines can particularly interact with the E-selectin protein, leading to increased drug accumulation at tumor locations [176,177]. Because of this, nanoparticles treated with sialic acid provide an excellent method by which to increase the efficacy of HCC detection and therapy. Fan et al. successfully achieved the early diagnosis of HCC by targeting E-selectin through sialic acid modified nanocomposites [94]. Due to the synergistic effect of the ferritin gene and SPIONs in nanocomposites, the capacity of T2-weighted images to increase MR contrast in tumor areas increased compared to that of previous preparations, and it also exhibited great biocompatibility. Exogenous and endogenous contrast enhancement have been effectively used in the diagnosis of HCC. Du and co-workers developed a temperature-sensitive micelle, which was coated with Gd-CuS nanoparticles and doxorubicin. The surface of the micelle was modified with sialic acid, and as such it showed a high affinity for HCC cells. Previous experiments demonstrate that the micelle could be highly accumulated in the tumor area through the sialic-acid-mediated targeted effect, and could trigger the instantaneous release of internal drugs under in vitro NIR irradiation, so as to achieve MR/photoacoustic dual-mode imaging and the combined treatment of chemotherapy and photothermal treatment, thereby effectively inhibiting cancer cells and achieving accurate imaging [95].

In addition to labeling HCC cells, the labeling of immune cells, such as NK cells, has recently been reported. Sim et al. synthesized and fabricated a magnetic nanocomplex with hyaluronic acid, protamine, and ferumoxytol, which exhibited efficient affinity with NK cells. The activation of NK cells was effectively realized with an exogenous magnetic field, which promoted the generation and secretion of the lytic granules of NK cells. The inhibition of the in situ tumor was successfully achieved by catheter infusion, and ideal therapeutic effects and MR imaging were achieved [178].

## 5. Conclusions and Perspectives

HCC is still a malignant tumor with high incidence and mortality, and early diagnosis and treatment are core in improving the prognosis of patients with HCC. The early diagnosis of HCC largely depends on MRI. As mentioned above, MRI has a good soft-tissue resolution, which is the international standard method for the diagnosis of HCC. However, MRI is still insufficient in the diagnosis of some early small HCCs and malignant nodules, resulting in false negative results. With the continued exploration of the tumor microenvironment in HCC, many specific targets have been found to be overexpressed on the surface of HCC cells, which has important potential in its diagnosis and treatment. On the other hand, the molecular imaging probe has led to great progress in recent years, especially in cancer diagnosis and treatment. Therefore, taking MR imaging as the basic point, we summarized the recent progress regarding the molecular imaging targeting of various types of biomarkers on the surface of HCC cells to improve the diagnostic rate of early HCC. Another important factor for improving the prognosis of HCC patients is early treatment, and the integration of diagnosis and treatment can provide patients with maximum clinical benefits, which is also an important aspect of future medical development. Therefore, this paper also summarized the basic research surrounding diagnosis and treatment combination, so as to provide a reference for future research.

When constructing receptor-targeted probes, it is critical to identify the target receptor and a ligand with a high affinity for the receptor. Ligands can take on a variety of forms, including peptides, antibodies, and aptamers. Each of these forms has distinct characteristics that should be considered while developing probes. For example, peptides have an excellent tissue-penetration ability due to their tiny size, antibodies have superior antigen recognition, and aptamers have a strong recombination ability, allowing for a better likelihood of binding to the target receptor. Additionally, numerous ligands can be attached to a single nanoparticle to increase target selectivity. Combined with the current research results, both the selection of molecular targets and the feasibility of material synthesis for the treatment or imaging show the potential and great possibility of application. For example, multimodal targeted imaging technology may play an essential role in the clinically precise positioning of the tumor edge, and assist in the surgical resection and real-time evaluation of therapeutic effects in the future. To be specific, a patient can perform preoperative MR imaging evaluation through the injection of multimodal molecular probes (such as MR/NIRF dual-modal targeted probes). During the subsequent operation, intraoperative near-infrared imaging can be performed on the liver with an NIR instrument. The tumor tissue can be excited by near-infrared light to accurately display its shape and distinguish it from normal liver parenchyma. The combination of the sectional anatomy information provided by MR imaging with the tumor visualization information obtained using intraoperative near-infrared imaging can accurately provide the information of tumor size, location, number and edge, so as to guide the intraoperative tumor resection and real-time evaluation of the resection range. In addition to simple diagnostic probes, diagnosis and treatment integration probes are also an important direction for future development. Nanoprobes, when combined with other functional materials and drugs, have the potential to be used in imaging-guided synergetic HCC therapy, such as MRI-guided photothermal treatment, and this integration probe is also consistent with the notion of precision medicine. However, despite the promising results that have been published in this sector, there are still certain obstacles that need to be overcome before those probes may be used in clinical settings in the future.

Currently, the most significant barrier to clinical application is the potential for long-term security concerns associated with these nanomaterials, particularly those that are nonbiodegradable and thus remain in the body for an extended period after being administered. Most previous experiments focused on targeting and imaging specificity, as well as short-term security. However, more experimental data are needed to verify the metabolism of these nanomaterials after imaging and treatment, and to evaluate whether irreversible damage will be caused to other organs, especially in liver and kidney metabolism. Hence, although most investigations in this review have established that the as-synthesized materials have outstanding biocompatibility, their long-term chronic toxic effects have yet to be comprehensively studied in greater detail. In addition, the stability of the combination of paramagnetic materials with probes needs to be evaluated to avoid tissue toxicity due to the shedding of substances such as Gd^3+^. Thirdly, the surface-modification process of nanomaterials is almost concentrated on the traditional physical and chemical methods, although researchers are trying to avoid steps that may lead to biotoxicity during the reaction, further improvements or alternative methods are still needed. Hence, we can explore other better modification approaches. For example, recent studies have found that researchers could extract biomineralized magnetic nanoparticles (magnetosomes) from magnetotactic bacteria [179,180]. The magnetosome protein located on the inner membrane of magnetotactic bacteria can mediate the depression of the inner membrane and polymerize the magnetite uptake by magnetotactic bacteria. Through the process of biomineralization, the biomodification (encapsulated by bacterial bio-membrane) of the formed polymeric magnetite was carried out and magnetosomes were formed [180]. Therefore, compared with the traditional surface-modification methods using physical and chemical processes, this innovative biological modification method shows ideal biocompatibility and safety, largely avoids possible biological toxicity and it may act as a feasible method in the future with good magnetic properties [181]. Fourthly, the expression of HCC-related biomarkers will be reduced due to the necrosis of a large number of tumor cells after treatment. Whether molecular imaging can continue to be used for evaluation after treatment needs to be discussed. Finally, almost all studies are limited to the level of cells and animals. Although it has shown promising research results, there are huge differences in the physiological and biochemical levels between humans and animals. The application of these results to the clinical setting requires further research.

## Figures and Tables

**Table 1:** Comparison of characteristics of passive delivery and active targeted delivery.

	Categories	Passive Delivery	Active Targeted Delivery
Properties			
The difficulty of preparation		Simple	Relatively complex
Intracellularization		Weak	Strong
Dosage administration		High	Low
Normal liver tissue-related toxicity		Relatively large	Almost no effect
Drug distribution in tumor tissue		Unevenly	Uniformly
Drug release		Uncontrollable	Controllable
Evaluation of therapeutic effect		Hard to predict	Predictable
Tumor targeted imaging		Poor imaging effect	Excellent imaging effect

**Table 2:** Summary of targeted-delivery strategy based on overexpressed receptors in HCC mentioned in this review.

Receptors	Matching Ligands	Theranostic Agents	Delivery Vehicles	Ref.
AFP	Antibody	Gd ion, paclitaxel	Polymeric micelle	[43]
		USPION	Surface modified USPION	[44,45]
		Cy7 fluorescent dye, Fe_3_O_4_	Liposome	[46]
GPC-3	Peptide	Gd ion	WS_2_-PEG	[47]
		FeSe_2_	FeSe_2_-PEG	[48]
		Fe_3_O_4_, Au, Cy5.5	Fe_3_O_4_ core/Au shell nanocomplex	[49]
		USPION	Surface modified USPION	[50]
	Aptamer	USPION	Surface modified USPION	[51]
	Antibody	Fe_3_O_4_	Semiconducting polymers	[52]
		SPION, sorafenib	Micelle	[53]
		SPION	Surface modified SPION	[54]
		PBNP	PBNP	[55]
Folate Receptor	Folic acid	SiRNA, SPION	Polymeric micelle	[56]
		Gd ion	Porphyrin metal-organic frameworks	[57]
		Gd ion	Phase transition nanodroplets	[58]
		Triptolide	Folate modified triptolide	[59]
		Fe_3_O_4_, arsenic trioxide	Mesoporous silica nanoparticles	[60]
		SPION, sorafenib	PLGA-PEG	[61]
		SiRNA, SPION	Amylose	[62]
		magneto-gold nanoparticle	Surface modified magneto-gold nanoparticle	[63]
		Magnetite nanoparticle	polydopamine-coated magnetite nanoparticles	[64]
		CuFeSe_2_	CuFeSe_2_-PEG	[65]
VEGF/VEGFR	Peptide	Gd ion	VEGF-Gd chelates	[66]
	Antibody	Gd ion	Polymeric nanoparticle	[67]
		SPION	Polymeric micelle	[68]
		Gd ion	Biotin-PLL-DTPA	[69]
		Sorafenib, Gd ion	Multiblock polymer	[70]
Integrin	Peptide	Gd ion	Peptide	[71]
		Fe_3_O_4_	Fe_3_O_4_ nanoparticles	[72,73]
		Gadolinium arsenate	Gadolinium arsenate nanoparticles	[74]
		SiRNA, SPION	Polymeric nanoparticle	[75]
		Fe_3_O_4_, doxorubicin	Polylysine dendrimer	[76]
		Gd ion, SPION	SPION nanoparticles	[77]
		NIRF probe, SPION	SPION nanoparticles	[78]
Endoglin	Aptamer	Cy5.5, Fe_3_O_4_	Magnetic-fluorescent endoglin aptamer nanoprobe	[79]
		Gd ion, NIR fluorophore	Dendrimer	[80]
		Fe_3_O_4_	Carboxymethyl chitosan nanoparticles	[81]
ASGP-R	Galactose	Fe_3_O_4_, NIRF probe	Lipid shell	[82]
	Lactobionic acid	Au nanoparticles, ICG, Gd (Ⅲ)-DOTA	Au nanoparticles @ polydopamine	[83]
		MicroRNA-99a	PLGA nanoparticles	[84]
		Manganese ferrite nanoparticle	Manganese ferrite nanoparticles	[85]
CD44	Hyaluronic acid	Au nanoparticles, Mn ion	Dendrimer	[86]
		Gd ion	Hyaluronic acid -DTPA-Gd	[87]
		Doxorubicin, SPION	Copolymers	[88]
	Antibody	Nd^3+^	Upconversion nanoparticle	[89]
CD90	Antibody	17-AAG, thermosensitive magnetoliposomes	Thermosensitive magnetoliposomes	[90]
		Fe_3_O_4_	Thermosensitive magnetoliposomes	[91]
EGFR	Peptide	USPION	Surface modified USPION	[92]
		SPION	Chitosan oligosaccharide micelle	[93]
E-selectin	Salic acid	Reporter gene, SPION	Mesoporous polydopamine	[94]
		Gd-CuS nanoparticle, Doxorubicin	Polymeric micelles	[95]

Abbreviations: AFP, alpha-fetoprotein; ASGP-R, asialoglycoprotein receptor; DTPA, diethylene triamine pentacetate acid; DOTA, 1,4,7,10-tetraazacyclododecane-*N*,*N*′,*N*,*N*′-tetraacetic acid; EGFR, epidermal growth factor receptor; GPC-3, glypican-3; ICG, indocyanine green; NIRF, near-infrared fluorescence; PEG, polyethylene glycol; PLL, poly-l-lysine; PLGA, poly(lactic-co-glycolic acid); PBNP, Prussian blue nanoparticle; SPION, superparamagnetic iron oxide nanoparticle; USPION, ultra-small superparamagnetic iron oxide nanoparticle; VEGF, vascular endothelial growth factor; VEGFR, vascular endothelial growth factor receptor; 17-AAG, 17-allylamino-17-demethoxygeldanamycin.

**Table 3:** The advantages and disadvantages of common ligand types.

	Categories	Antibody	Peptide	Aptamer	Small Molecule
Characteristics					
Advantages		Mature technology	Non-immunogenicity	Strong recombination ability	Non-immunogenicity
		Wide application	Strong tissue penetration	High affinity and specificity	Conjugation chemistry
		High stability	Strong editability	Three-dimensional structure	High stability
		Mass production	Self-assembly	Non-immunogenicity	High affinity
		High affinity and specificity	High preparation cost	Overcome the limitation of cell lines and animal needs	Easy to obtain
			Simple organization	High stability	Good safety
			Low molecular weight	Mass production	Low cost
			High stability	Small volume	
Disadvantages		Large molecular weight	strictly screened	Labor intensive	Rapid clearance in vivo
		Poor tissue penetration	Labor intensive	Time consuming	off-target effect
		Easily phagocytized nonspecifically	Time consuming	Vulnerable affinity and specificity	
		Difficult to fold	Difficulties in screening ideal peptides	Consume substantial funds and resources	
		Immunogenicity			
		Long metabolic cycle			
		High preparation cost			

## Data Availability

Not applicable.
